# Loneliness, social isolation, and pain following the COVID-19 outbreak: data from a nationwide internet survey in Japan

**DOI:** 10.1038/s41598-021-97136-3

**Published:** 2021-09-20

**Authors:** Keiko Yamada, Kenta Wakaizumi, Yasuhiko Kubota, Hiroshi Murayama, Takahiro Tabuchi

**Affiliations:** 1 Department of Psychology, McGill University, 2001 McGill College Avenue, Montreal, QC H3A 1G1 Canada; 2 Department of Anesthesiology and Pain Medicine, Juntendo University Faculty of Medicine, 2-1-1 Hongo, Bunkyo-ku, Tokyo 113-8421 Japan; 3 Department of Anesthesiology, Keio University School of Medicine, 35 Shinanomachi, Shinjuku-ku, Tokyo 160-8582 Japan; 4 Interdisciplinary Pain Center, Keio University Hospital, 35 Shinanomachi, Shinjuku-ku, Tokyo 160-8582 Japan; 5 Osaka Center for Cancer and Cardiovascular Diseases Prevention, 1-6-107 Morinomiya, Jyoto-ku, Osaka 536-8588 Japan; 6 Research Team for Social Participation and Community Health, Tokyo Metropolitan Institute of Gerontology, 35-2 Sakae-cho, Itabashi-ku, Tokyo 173-0015 Japan; 7 Cancer Control Center, Osaka International Cancer Institute, 3-1-69 Otemae, Chuo-ku, Osaka 541-8567 Japan

**Keywords:** Medical research, Neurology, Rheumatology, Risk factors

## Abstract

The aim of cross-sectional study was to investigate the association between loneliness, increased social isolation, and pain following the COVID-19 outbreak. A total of 25,482 participants, aged 15–79 years, were assessed using an internet survey; the University of California, Los Angeles Loneliness Scale (Version 3), Short Form 3-item (UCLA-LS3-SF3) was used to assess loneliness, and a modified item of the UCLA-LS3-SF3 was used to measure the perception of increased social isolation during the pandemic. The outcome measures included the prevalence/incidence of pain (i.e., headache, neck or shoulder pain, upper limb pain, low back pain, and leg pain), pain intensity, and the prevalence of past/present chronic pain. Pain intensity was measured by the pain/discomfort item of the 5-level version of the EuroQol 5 Dimension scale. Odds ratios of pain prevalence/incidence and past/present chronic pain prevalence according to the UCLA-LS3-SF3 scoring groups (tertiles) and the frequency of the perceived increase in social isolation (categories 1–5) were calculated using multinomial logistic regression analysis. The mean pain intensity values among different loneliness and social isolation levels were tested using an analysis of covariance. Increased loneliness and the severity of the perceived social isolation were positively associated with the prevalence/incidence of pain, pain intensity, and the prevalence of past/present chronic pain.

## Introduction

Increased loneliness and social isolation due to the implementation of physical distancing measures and travel restrictions to prevent transmission during the coronavirus disease 2019 (COVID-19) pandemic have adversely affected physical and mental health throughout the world^1–4^. Loneliness and social isolation are great determinants of health and are associated with quality of life^5^ measures and psychological disorders, such as depression^6^, as well as with physical diseases such as cardiovascular diseases^7^ and increased blood pressure^8^. Moreover, a systematic review reported that loneliness and social isolation were risk factors of early mortality, with an increased likelihood of death ranging from 26 to 29%^9^.

Loneliness and social isolation are also well-known psychosocial risk factors for the exacerbation of pain^10,11^. A previous epidemiological study using data from the English Longitudinal Study of Ageing (ELSA) cohort reported that loneliness was associated with the higher prevalence of musculoskeletal pain^12^. Other studies demonstrated that loneliness is strongly associated with pain^10,13,14^, and social isolation predicted pain interference^11^. Therefore, a worsening of psychological stress due to the increased loneliness and social isolation experienced during the COVID-19 pandemic may contribute to the onset of pain^15^. Moreover, increased loneliness and social isolation during the COVID-19 pandemic may have a large impact on individuals with chronic pain who have a low tolerance for psychological stress^15–17^. However, there is lack of direct evidence of an association between pain and increased loneliness and social isolation following the COVID-19 outbreak; this highlights the need for a large population-based study to address an important and urgent health issue.

Loneliness is considered to be felt sad and alone, and no consensus definition currently exists. Based on a review of the literature, Peplau & Perlman (1982) offered the following definition: “the aversive state experienced when a discrepancy exists between the interpersonal relationships one wishes to have, and those that one perceives they currently have;” thus, it is an emotionally unpleasant experience^18,19^. In addition, two distinct types of loneliness exist, including *emotional loneliness* and *social loneliness*^18^. *Emotional loneliness* is defined as “results from the lack of a close, intimate attachment to another person” and *social loneliness* is defined as “results from the lack of a network of social relationships in which the person is part of a group of friends who share common interests and activities”^18^. On the other hand, social isolation is structural and can be described objectively based on the characteristics of the situation^20^; it is often distinguished from loneliness in an academic context, although loneliness and social isolation are sometimes used collectively, as the concept of social isolation partially overlaps with the concept of *social loneliness*^18^.

Therefore, the aims of the present study were to investigate the associations between the loneliness and increased social isolation experienced during the COVID-19 pandemic and (1) the prevalence/incidence of pain (i.e., headache, neck or shoulder pain, upper arm pain, low back pain, and leg pain), (2) pain intensity, and (3) the prevalence of past/present chronic pain (pain lasting ≥ 3 months) using data from a large cross-sectional survey of social and health variables related to COVID-19 in Japan.

## Methods

### Study design

The Japan COVID-19 and Society Internet Survey (JACSIS) was designed to investigate the social and health situation related to the COVID-19 pandemic using an epidemiological approach. From August 25 to September 30 in 2020, a total of 28,000 respondents, aged 15–79 years, were selected from 224,389 candidates who received an e-mail invitation among the approximately 2.2 million panelists registered with a Japanese internet survey agency (Rakuten Insight, Inc., Tokyo, Japan https://in.m.aipsurveys.com). A random sampling method was used to recruit participants using a computer algorithm; the sample was representative of the official Japanese demographic composition as of October 1, 2019 based on categories of age, sex, and living area (i.e., prefecture). All participants provided web-based informed consent before responding to the online self-report questionnaire. More specific information pertaining to the JACSIS, along with a detailed description of the method used to recruit participants is described in the Supplementary Methods. The present study employed a cross-sectional design intended to detect changes in biopsychosocial and socioeconomic factors before and after the COVID-19 outbreak.

### Study population

Of 28,000 participants in the JACSIS, we analyzed data from 25,482 participants (12,809 women and 12,673 men) after excluding 2518 participants who provided invalid responses or met other exclusion criteria; these measures to consistently validate the data quality were performed as described in previous studies^21,22^. The participants with invalid responses were detected through the use of a dummy item, which stated, “Please choose the option second from the bottom.” Participants who selected any option other than the one indicated were excluded (n = 1955). Participants using “all” recreational substances and medications (i.e., sleeping pills, anxiolytic agents, legal/illegal opioids, cannabis, cocaine, etc.) were excluded, as were those with “all” chronic diseases (i.e., diabetes, asthma, stroke, ischemic heart disease, cancer, mental disease, etc.); this resulted in the exclusion of 422 and 141 participants, respectively. The characteristics of the 25,482 participants who were included in the final analysis are shown in Supplementary Table 1.

### Main outcome measures

#### Loneliness during the COVID-19 pandemic

A Japanese version of the University of California, Los Angeles Loneliness Scale (Version 3), Short Form 3-item (UCLA-LS3-SF3) was used to assess loneliness; the scale was originally developed in English, although both the English and Japanese versions have previously been determined to be valid and reliable^23,24^. The items were as follows: (1) “How often do you feel that you lack companionship?”; (2) “How often do you feel left out?”; and (3) “How often do you feel isolated from others?” Participants were asked to rate the frequency in which these feelings were experienced over the past 30 days using a five-point scale, ranging from 1 to 5 [1 (Never), 2 (Rarely), 3 (Sometimes), 4 (Often), 5 (Always)]; this was modified from the original UCLA-LS3-SF3, which used a four-point frequency scale^23,24^. The total UCLA-LS3-SF3 scores in the current study ranged from 3 to 15, with higher scores indicating more severe loneliness^23,24^. We defined those in the first tertile (3 points), the second tertile (4–5 points), and the third tertile (6–15 points) of the UCLA-LS3-SF3 scores as having ‘no loneliness’, ‘mild loneliness’, and ‘moderate-to-severe loneliness’, respectively. The Cronbach’s alpha value of the internal consistency for the three items was 0.93.

#### Perception of increased social isolation during the COVID-19 pandemic

The following single question was utilized to measure the perception of increased social isolation during the COVID-19 pandemic: “How often do you feel increased isolation from others compared with how you felt before the COVID-19 outbreak (prior to January 2020)?” The ratings were based on the same five-point frequency scale as the UCLA-LS3-SF3, with responses scored as follows: never (1), rarely (2), sometimes (3), often (4), or always (5). This question was an adaptation of the third item of the UCLA-LS3-SF3; it was modified for the JACSIS to better capture changes in the perceived social isolation between the pre- and post-COVID-19 outbreak timepoints.

#### Pain

##### Prevalence and incidence of pain

Participants were asked if they had a headache, neck or shoulder pain, upper limb pain, low back pain, or leg pain; for each type of pain, the responses were categorized as follows: “none”, “yes, it developed before the COVID-19 outbreak”, or “yes, it developed during the COVID-19 pandemic.” Based on these responses, the participants were classified based on one of the following three categories: without pain, the presence of pain since before the COVID-19 outbreak, and pain beginning during the COVID-19 pandemic.

##### Pain intensity

Pain intensity was assessed as a continuous variable based on the pain/discomfort item of a Japanese adaptation of the 5-level version of the EuroQol 5 Dimension (EQ5D-5L) scale^25^. The item was phrased as follows: “Please choose the option that best describes your health today.” Participants rated their present pain/discomfort on a scale of 1–5 based on the following descriptions: (1) I have no pain or discomfort, (2) I have slight pain or discomfort, (3) I have moderate pain or discomfort, (4) I have severe pain or discomfort, or (5) I have extreme pain or discomfort.

##### Past and present chronic pain

Participants were asked if they had experienced chronic pain for three months or more; the responses were based on the following categorical options: “none,” “I have a history of chronic pain but have already recovered”, “yes, receiving treatment”, or “yes, without treatment.” The data of individuals who had a history of chronic pain but had already recovered (i.e., history of past chronic pain) were collected because those individuals may have had a lower tolerance for psychosocial stressors, such as loneliness and social isolation, similar to the individuals who reported that they were currently experiencing chronic pain. We considered the latter two categories (having chronic pain with or without treatment) as history of present chronic pain, and we classified the participants into the following three categories: those without chronic pain, those with a history of past chronic pain, and those with a history of present chronic pain (currently experiencing chronic pain).

### Potential confounders

Potential confounders were mainly adapted from previous population-based cohort studies that have examined the associations between psychosocial factors and pain experience^26–28^. These included demographic factors, socioeconomic factors, lifestyle factors, and a history of diseases related to the explanatory and outcome variables.

#### Demographic factors

We collected data on age (15–19, 20–29, 30–39, 40–49, 50–59, 60–69, or 70–79 years), sex (women or men), and body mass index (quintiles).

#### Socioeconomic factors

We collected data on the level of education completed (less than high school, high school, vocational school, junior or technical college, university, graduate school, or other), marital status (married or common-law, single, divorced, or widowed), whether the individual was living alone (yes or no), employment status (company executive, owner of a family-operated business, employee of a family-operated business, management-level employee, full-time employee, contract employee, part-time employee/on-the-side worker, student, retired, full-time homemaker, or unemployed), and equivalized income (categorized into quintiles).

Equivalized income was calculated by dividing the median value of the multiple-choice annual household income before-tax by the square root of the number of people living in the household. We defined poverty as an annual equivalized income of less than 1.22 million JPY, which was the poverty line in 2018 as defined by the Organisation for Economic Co-operation and Development^25^.

#### Life style

We collected data on smoking status (non-smoker, ex-smoker, or current smoker), alcohol consumption (never, ex-drinker, social drinker, < 23 g per day, 23–45 g per day, or ≥ 46 g per day), changes in physical activity before and after the COVID-19 outbreak (decreased, no change, or increased), and sleep duration (< 4 h, 4–5 h, 6–7 h, 8–9 h, ≥ 10 h, or hard to respond/unsure). We defined any individual with alcohol consumption ≥ 46 g per day as a heavy drinker.

#### History of mental diseases

We collected data on the history of depression (categorized as none, a history of depression but already recovered, or comorbid depression) and the history of mental illnesses other than depression (categorized as none, a history of mental illness other than depression but already recovered, or comorbid mental illness other than depression).

### Statistical analysis

First, we determined the *P* for trend of the means and proportions of potential confounders according to the severity of loneliness based on the UCLA-LS3-SF3 scoring groups and the frequency in which participants felt increased social isolation during the COVID-19 pandemic using a general linear model.

Second, we examined the association of loneliness and increased social isolation and the prevalence/incidence of pain experienced during the COVID-19 pandemic. The odds ratios (ORs) of the prevalence and incidence of pain (i.e., headache, neck or shoulder pain, upper limb pain, low back pain, or leg pain) according to the UCLA-LS3-SF-3 scoring groups were calculated using a multinomial logistic regression model, with adjustment for potential confounders.

Third, the overall adjusted mean values of pain intensity for all participants and for individuals with any pain symptoms according to the UCLA-LS3-SF3 scoring groups and the frequency in which feelings of increased social isolation were experienced during the COVID-19 pandemic were tested using an analysis of covariance, with Dunnett’s test. Individuals with any pain symptoms were defined as those with headache, neck/shoulder pain, upper limb pain, low back pain, leg pain, or the presence of chronic pain. *P* values were calculated to compare those reporting a lack of loneliness or those who did not feel an increase in social isolation during the COVID-19 pandemic at all to those of all the other categories.

Finally, we examined the association between the loneliness and increased social isolation felt during the COVID-19 pandemic and the prevalence of past and present chronic pain. The ORs of the prevalence of past and present chronic pain according to the UCLA-LS3-SF3 scoring groups and the frequency of feelings of increased social isolation were calculated using a multinomial logistic regression model, adjusted for potential confounders.

A multinomial logistic regression analysis is often used to estimate multiple categorical outcomes (i.e., the prevalence/incidence of pain and the prevalence of past/present chronic pain). In addition to the ORs, the *P* for trends according to the UCLA-LS3-SF3 scoring groups and the frequency of feelings of increased social isolation during the COVID-19 pandemic were calculated using a general linear model.

Model 1 was adjusted for age and sex, whereas Model 2 was adjusted for age, sex, level of education, marital status, living arrangement (living alone or not), employment status, equivalized income, smoking status, alcohol consumption, physical activity, sleep duration, history of depression, and history of mental illnesses other than depression.

Missing values of potential confounders were used as dummy variables. *P* values < 0.05 (two-tailed tests) were considered statistically significant. All statistical analyses were performed using Statistical Analysis Software (SAS), Version 9.4 (SAS Institute Inc., Cary, NC, USA).

### Ethical issues

All procedures were conducted in accordance with the ethical standards of the Helsinki Declaration of 1975, as revised in 2013. The study protocol was reviewed and approved by the Research Ethics Committee of the Osaka International Cancer Institute (approved on June 19, 2020; approval number 20084). The internet survey agency respected the Act on the Protection of Personal Information in Japan. All participants provided web-based informed consent before responding to the online questionnaire. This study was exempted from the obligation to obtain informed consent from the parents/guardians of minors under the age of 18 in Japan. The Ethical Guidelines for Medical and Health Research Involving Human Subjects enforced by Japan’s Ministry of Education, Culture, Sports, Science and Technology and Japan’s Ministry of Health, Labour and Welfare addressed, “when the research individuals have completed junior high school or another relevant schooling, or is 16 years or older, and is considered to have enough judgment concerning the research to be implemented on themselves, and the following matters are prescribed in the research protocol, and the chief executive of the research implementing entity approves to carry out the research after relevant ethical review committee deliberation, informed consent shall be obtained not from representative but from the said research subject. (1) The research to be implemented does not involve any invasiveness; and (2) Information concerning the implementation of the research, including the purpose of the research and how specimens or information will be handled, is made public, and opportunities to refuse that the research is commenced or continued on the research subject are ensured for persons who exercise parental authority over the said research subject and guardians of the minor^29^.” All participants completed junior high school, the present study did not involve any invasiveness, and the approval of the Research Ethics Committee of the Osaka International Cancer Institute for the study protocol was obtained as aforementioned. A credit point known as “Epoints”, which could be used for internet shopping and cash conversion, was provided to the participants as an incentive.

## Results

### Descriptive statistics

Of a total of 25,482 participants, 1033 (4.1%) and 639 (2.5%) participants reported feeling increased social isolation during the COVID-19 pandemic often or always, respectively. The prevalences of headache, neck or shoulder pain, upper limb pain, low back pain, and leg pain were 23.5%, 42.4%, 16.6%, 33.3%, and 17.8%, respectively. The incidences of headache, neck or shoulder pain, upper limb pain, low back pain, and leg pain after the start of the COVD-19 outbreak were 2.0%, 2.4%, 1.8%, 2.1%, 1.5%, respectively. In terms of pain intensity, a total of 7% reported experiencing moderate-to-extreme pain. A total of 6.3% of participants had a history of chronic pain but had already recovered (i.e., a history of past chronic pain), 3.9% had a history of present chronic pain but were receiving treatment, and 6.5% had a history of present chronic pain that was not being treated.

The mean values and proportions related to the characteristics of the participants according to their UCLA-LS3-SF3 scoring group and the frequency of feelings of increased social isolation are indicated in Table 1. Compared with participants who did not report experiencing loneliness (the first quartile of the UCLA-LS3-SF3 score), those within the second to fourth quartiles based on the UCLA-LS3-SF3 scores were more likely to be younger and female, to have a lower educational level, to be divorced, to live alone, to be experiencing poverty, to be current smokers, to have decreased their level of physical activity during the COVID-19 pandemic, to sleep less than six hours per night, to have comorbid depression, and to have comorbid mental illnesses other than depression; they were less likely to be obese and heavy drinkers. The prevalence of unemployment did not vary among the UCLA-LS3-SF3 scoring groups.

**Table 1:** Mean values and proportions for participant characteristics (n = 25,482).

	Total		Degree of loneliness							Frequency of feelings of increased social isolation during the COVID-19 pandemic										
			None (3 points)		Mild (4–5 points)		Moderate-to-severe (6–15 points)		*P* for trend	Never		Rarely		Sometimes		Often		Always		*P* for trend
	n = 25,482		n = 14,277		n = 3250		n = 7955			n = 18,168		n = 3188		n = 2454		n = 1033		n = 639		
	Mean	SD	Mean	SD	Mean	SD	Mean	SD		Mean	SD	Mean	SD	Mean	SD	Mean	SD	Mean	SD	
	n	(%)	n	(%)	n	(%)	n	(%)		n	(%)	n	(%)	n	(%)	n	(%)	n	(%)	
Age, years	48.8	17.3	51.5	17.3	50.3	17	43.3	16.2	< 0.001	50.7	17.1	48.1	17.3	42.2	16.3	38.5	15.5	38.2	14.4	< 0.001
Women	12,809	50.3	6794	47.6	1828	56.2	4187	52.6	< 0.001	8809	48.5	1778	55.8	1312	53.5	555	53.7	355	55.6	< 0.001
Body mass index ≥ 25 kg/m^2^	4910	19.3	2864	20.1	543	16.7	1503	18.9	< 0.001	3611	19.9	566	17.8	431	17.6	173	16.7	129	20.2	0.001
Less than high school graduate	1014	4.0	540	3.8	93	2.9	381	4.8	< 0.001	718	4.0	93	2.9	116	4.7	45	4.4	42	6.6	< 0.001
Divorced	1602	6.3	841	5.9	212	6.5	549	6.9	0.01	1107	6.1	206	6.5	179	7.3	60	5.8	50	7.8	0.07
Living alone	4997	19.6	2431	17.0	644	19.8	1922	24.2	< 0.001	3260	17.9	688	21.6	599	24.4	283	27.4	167	26.1	< 0.001
Unemployed	3015	11.8	1708	12.0	374	11.5	933	11.7	0.72	2266	12.5	346	10.9	219	8.9	85	8.2	99	15.5	< 0.001
Living in poverty	1393	5.5	649	4.5	156	4.8	588	7.4	< 0.001	888	4.9	178	5.6	174	7.1	80	7.7	73	11.4	< 0.001
Current smoker	4581	18.0	2546	17.8	530	16.3	1505	18.9	0.004	3239	17.8	570	17.9	447	18.2	215	20.8	110	17.2	0.18
Heavy drinker	2144	8.4	1284	9.0	263	8.1	597	7.5	< 0.001	1620	8.9	238	7.5	164	6.7	72	7.0	50	7.8	< 0.001
Decreased physical activity during COVID-19 pandemic	7358	28.9	3774	26.4	1022	31.4	2562	32.2	< 0.001	4712	25.9	1180	37.0	766	31.2	429	41.5	271	42.4	< 0.001
Sleep duration < 6 h per night	6639	26.1	3312	23.2	716	22.0	2611	32.8	< 0.001	4223	23.2	862	27.0	927	37.8	381	36.9	246	38.5	< 0.001
Comorbid depression	969	3.8	172	1.2	66	2.0	731	9.2	< 0.001	378	2.1	157	4.9	181	7.4	147	14.2	106	16.6	< 0.001
Comorbid mental illnesses other than depression	950	3.7	182	1.3	58	1.8	710	8.9	< 0.001	371	2.0	158	5.0	173	7.0	130	12.6	118	18.5	< 0.001

*COVID-19* coronavirus disease 2019, *SD* standard deviation.

Loneliness was measured using the Japanese version of the University of California, Los Angeles Loneliness Scale (Version 3), Short Form 3-item tool.

Poverty was defined as an equivalized income less than 1.22 million Japanese yen.

Individuals with alcohol consumption ≥ 46 g per day were defined as heavy drinkers.

*P* for trend values were calculated using a generalized linear model.

Compared to participants who did not report feeling an increase in social isolation, those who felt more socially isolated during the COVID-19 pandemic were more likely to be younger and obese, to have a lower educational level, to live alone, to be unemployed and living in poverty, to have decreased their level of physical activity during the COVID-19 pandemic, to sleep less than six hours per night, to have comorbid depression, and to have comorbid mental illnesses other than depression; they were also less likely to be heavy drinkers. The prevalences of those who were divorced and those who currently smoked did not vary based on the perceived increase in social isolation.

### Prevalence/ incidence of pain

**Figure 1:**
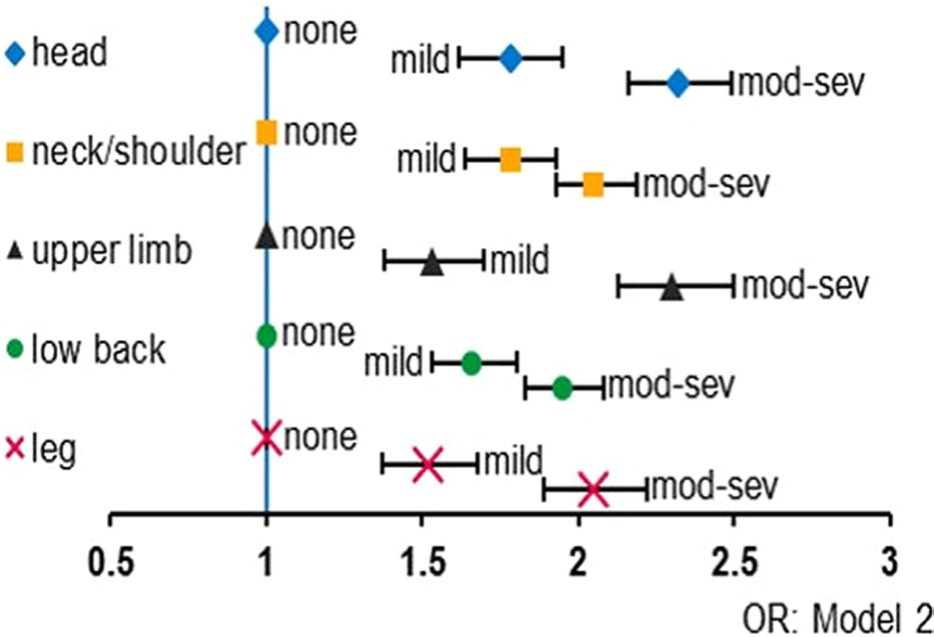
Degree of loneliness and pain symptoms since before the COVID-19 outbreak. Odds ratios of each pain symptom in model 2 by the degree of loneliness compared to none of the loneliness were indicated. X-axis indicated odds ratio. Bars indicated 95% confidence intervals. Abbreviation: *mod-sev* moderate-to-severe, *OR* odds ratio.

**Figure 2:**
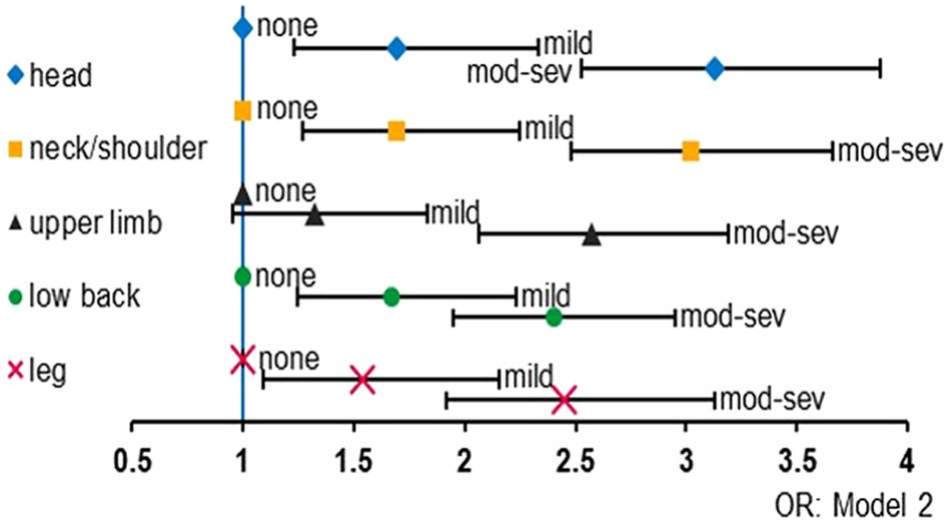
Degree of loneliness and pain symptoms developed during the COVID-19 pandemic. Odds ratios of each pain symptom in model 2 by the degree of loneliness compared to none of the loneliness were indicated. X-axis indicated odds ratio. Bars indicated 95% confidence intervals. Abbreviations: *mod-sev* moderate-to-severe, *OR* odds ratio.

**Figure 3:**
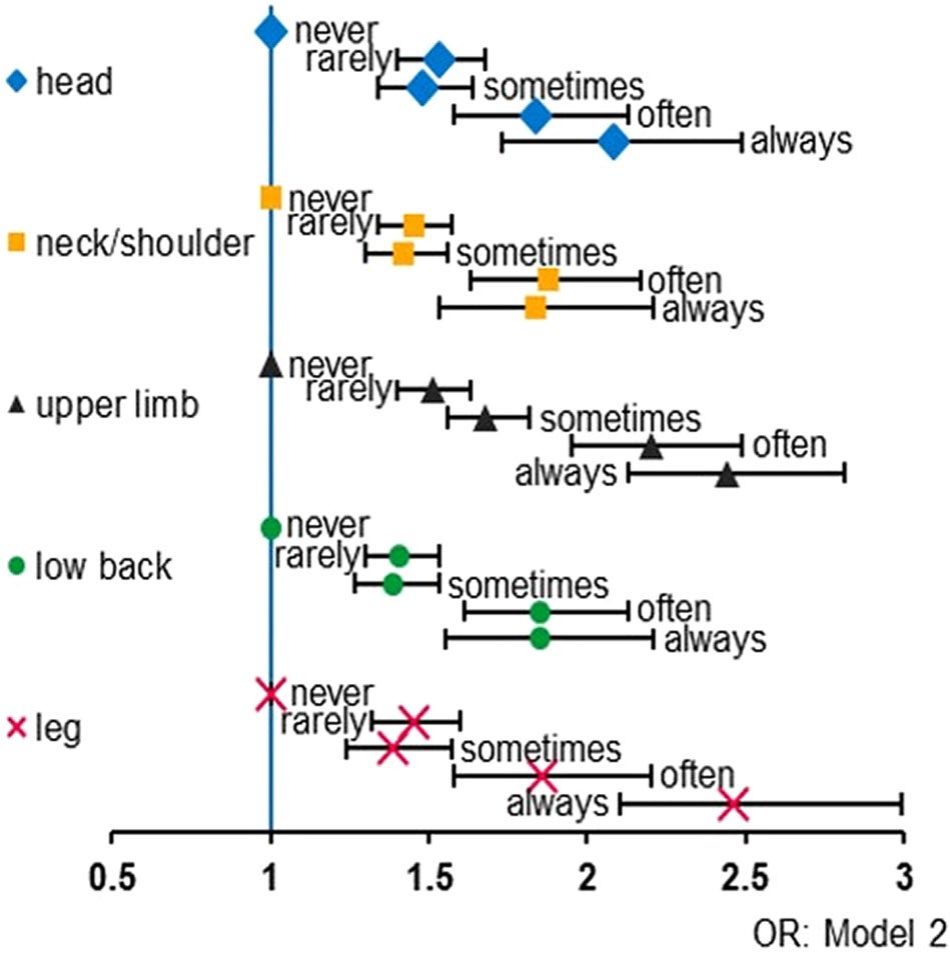
Frequency of feelings of social isolation and pain symptoms since before the COVID-19 outbreak. Odds ratios of each pain symptom in model 2 according to the frequency of feelings of social isolation compared to never felt feelings of social isolation were indicated. X-axis indicated odds ratio. Bars indicated 95% confidence intervals. Abbreviation: *OR* odds ratio.

**Figure 4:**
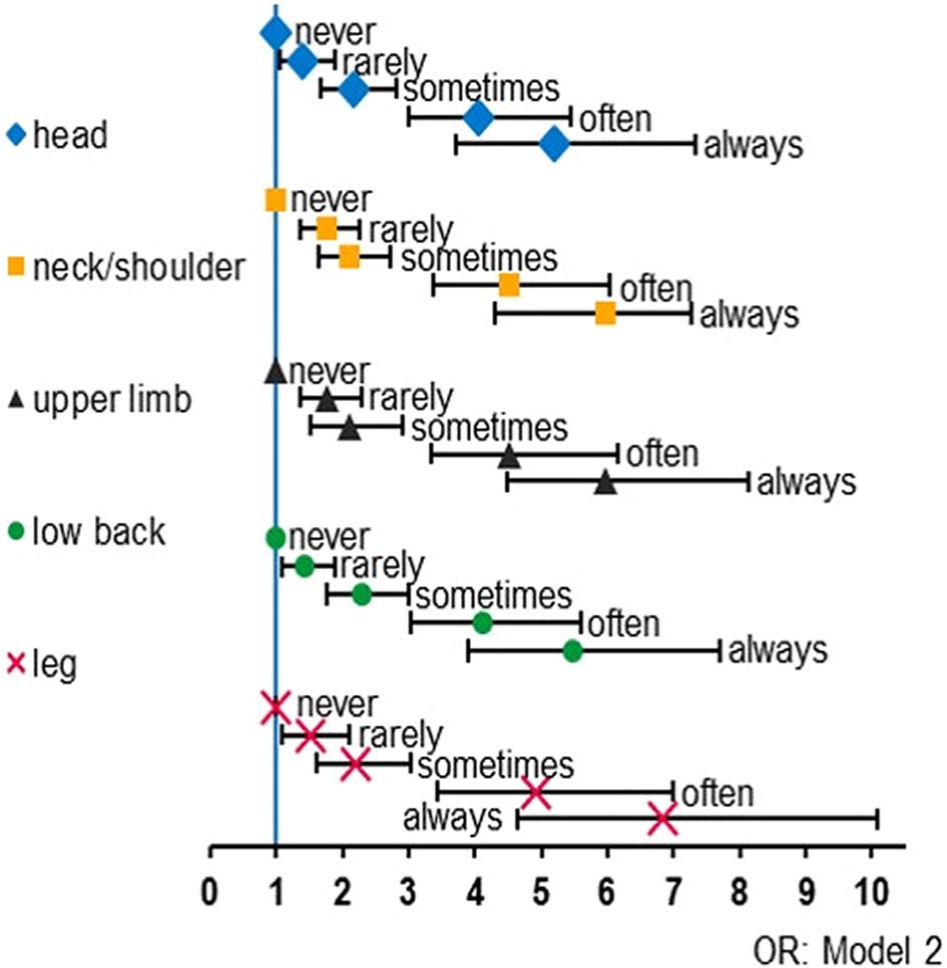
Frequency of feelings of social isolation and pain symptoms developed during the COVID-19 pandemic. Odds ratios of each pain symptom in model 2 according to the frequency of feelings of social isolation compared to never felt feelings of social isolation were indicated. X-axis indicated odds ratio. Bars indicated 95% confidence intervals. Abbreviation: *OR* odds ratio.

Table 2, Figs. 1, 2, 3, and 4 indicate that both loneliness and the perception of increased social isolation during the pandemic were positively associated with the prevalence and incidence of all types of pain (i.e., headache, neck or shoulder pain, upper limb pain, low back pain, and leg pain).

**Table 2:** The association of loneliness and a feeling of increased social isolation with the prevalence/incidence of pain during the COVID-19 pandemic.

	Degree of loneliness					
	None (3 points) n = 14,277	Mild (4–5 points) n = 3250	Moderate-to-Severe (6–15 points) n = 7955	*P* for trend		
**Headache**						
Number with headache since before the COVID-19 outbreak	2251	888	2848			
Number with headache developed during the COVID-19 pandemic	150	54	306			
**Model 1**		**OR (95% CI)**	**OR (95% CI)**			
Headache since before the COVID-19 outbreak vs. without headache	1	1.92 (1.75–2.11)***	2.65 (2.47–2.83)***	< 0.001		
Headache developed during the COVID-19 pandemic vs. without headache	1	1.82 (1.33–2.49)***	4.10 (3.31–5.01)***	< 0.001		
**Model 2**						
Headache since before the COVID-19 outbreak vs. without headache	1	1.78 (1.62–1.95)***	2.32 (2.16–2.49)***	< 0.001		
Headache developed during the COVID-19 pandemic vs. without headache	1	1.69 (1.23–2.33)**	3.13 (2.53–3.88)***	< 0.001		
**Neck or shoulder pain**						
Number with neck or shoulder pain since before the COVID-19 outbreak	4972	1665	4166			
Number with neck or shoulder pain developed during the COVID-19 pandemic	204	65	333			
**Model 1**		**OR (95% CI)**	**OR (95% CI)**			
Neck or shoulder pain since before the COVID-19 outbreak vs. without neck or shoulder pain	1	1.94 (1.79–2.10)***	2.21 (2.09–2.35)***	< 0.001		
Neck or shoulder pain developed during the COVID-19 pandemic vs. without neck or shoulder pain	1	1.86 (1.40–2.47)***	3.84 (3.20–4.60)***	< 0.001		
**Model 2**						
Neck or shoulder pain since before the COVID-19 outbreak vs. without neck or shoulder pain	1	1.78 (1.64–1.93)***	2.05 (1.93–2.19)***	< 0.001		
Neck or shoulder pain developed during the COVID-19 pandemic vs. without neck or shoulder pain	1	1.69 (1.27–2.25)***	3.02 (2.48–3.66)***	< 0.001		
**Upper limb pain**						
Number with upper limb pain since before the COVID-19 outbreak	1736	596	1904			
Number with upper limb pain developed during the COVID-19 pandemic	161	49	253			
**Model 1**		**OR (95% CI)**	**OR (95% CI)**			
Upper limb pain since before the COVID-19 outbreak vs. without upper limb pain	1	1.64 (1.48–1.82)***	2.71 (2.52–2.93)***	< 0.001		
Upper limb pain developed during the COVID-19 pandemic vs. without upper limb pain	1	1.42 (1.03–1.96)*	3.28 (2.67–4.03)***	< 0.001		
**Model 2**						
Upper limb pain since before the COVID-19 outbreak vs. without upper limb pain	1	1.53 (1.38–1.70)***	2.30 (2.13–2.50)***	< 0.001		
Upper limb pain developed during the COVID-19 pandemic vs. without upper limb pain	1	1.32 (0.95–1.83)	2.57 (2.06–3.19)***	< 0.001		
**Low back pain**						
Number with low back pain since before the COVID-19 outbreak	3942	1298	3247			
Number with low back pain developed during the COVID-19 pandemic	194	65	275			
**Model 1**		**OR (95% CI)**	**OR (95% CI)**			
Low back pain since before the COVID-19 outbreak vs. without low back pain	1	1.79 (1.65–1.94)***	2.11 (1.99–2.24)***	< 0.001		
Low back pain developed during the COVID-19 pandemic vs. without low back pain	1	1.78 (1.36–2.41)***	3.14 (2.59–3.81)***	< 0.001		
**Model 2**						
Low back pain since before the COVID-19 outbreak vs. without low back pain	1	1.66 (1.53–1.80)***	1.95 (1.83–2.08)***	< 0.001		
Low back pain developed during the COVID-19 pandemic vs. without low back pain	1	1.67 (1.25–2.23)***	2.40 (1.95–2.95)***	< 0.001		
**Leg pain**						
Number with leg pain since before the COVID-19 outbreak	2030	666	1828			
Number with leg pain developed during the COVID-19 pandemic	136	46	195			
**Model 1**		**OR (95% CI)**	**OR (95% CI)**			
Leg pain since before the COVID-19 outbreak vs. without leg pain	1	1.61 (1.45–1.77)***	2.37 (2.20–2.55)***	< 0.001		
Leg pain developed during the COVID-19 pandemic vs. without leg pain	1	1.60 (1.14–2.24)**	3.23 (2.57–4.06)***	< 0.001		
**Model 2**						
Leg pain since before the COVID-19 outbreak vs. without leg pain	1	1.52 (1.37–1.68)***	2.05 (1.89–2.22)***	< 0.001		
Leg pain developed during the COVID-19 pandemic vs. without leg pain	1	1.54 (1.09–2.16)*	2.45 (1.92–3.13)***	< 0.001		

	Frequency of feelings of increased social isolation during the COVID-19 pandemic					
	Never n = 18,168	Rarely n = 3188	Sometimes n = 2454	Often n = 1033	Always n = 639	*P* for trend
**Headache**						
Number with headache since before the COVID-19 outbreak	3586	966	768	398	269	
Number with headache developed during the COVID-19 pandemic	231	63	88	74	54	
**Model 1**		**OR (95% CI)**	**OR (95% CI)**	**OR (95% CI)**	**OR (95% CI)**	
Headache since before the COVID-19 outbreak vs. without headache	1	1.65 (1.51–1.80)***	1.55 (1.41–1.71)***	2.15 (1.87–2.47)***	2.55 (2.15–3.03)***	< 0.001
Headache developed during the COVID-19 pandemic vs. without headache	1	1.69 (1.27–2.24)***	2.70 (2.09–3.48)***	5.93 (4.48–7.85)***	7.66 (5.56–10.57)***	< 0.001
**Model 2**						
Headache since before the COVID-19 outbreak vs. without headache	1	1.53 (1.40–1.68)***	1.48 (1.34–1.64)***	1.84 (1.58–2.13)***	2.08 (1.73–2.49)***	< 0.001
Headache developed during the COVID-19 outbreak vs. without headache	1	1.41 (1.06–1.88)*	2.16 (1.66–2.81)***	4.04 (3.00–5.45)***	5.21 (3.71–7.33)***	< 0.001
**Neck or shoulder pain**						
Number with neck or shoulder pain since before the COVID-19 outbreak	7162	1596	1152	554	339	
Number with neck or shoulder pain developed during the COVID-19 pandemic	282	88	91	79	62	
**Model 1**		**OR (95% CI)**	**OR (95% CI)**	**OR (95% CI)**	**OR (95% CI)**	
Neck or shoulder pain since before the COVID-19 outbreak vs. without neck or shoulder pain	1	1.54 (1.42–1.66)***	1.37 (1.26–1.49)***	2.05 (1.79–2.35)***	2.09 (1.76–2.48)***	< 0.001
Neck or shoulder pain developed during the COVID-19 pandemic vs. without neck or shoulder pain	1	2.13 (1.66–2.72)***	2.56 (2.00–3.27)***	6.46 (4.91–8.50)***	8.47 (6.22–11.53)***	< 0.001
**Model 2**						
Neck or shoulder pain since before the COVID-19 outbreak vs. without neck or shoulder pain	1	1.45 (1.34–1.57)***	1.42 (1.30–1.56)***	1.88 (1.63–2.17)***	1.84 (1.53–2.21)***	< 0.001
Neck or shoulder pain developed during the COVID-19 pandemic vs. without neck or shoulder pain	1	1.77 (1.38–2.27)***	2.11 (1.63–2.73)***	4.52 (3.37–6.04)***	5.97 (4.31–8.27)***	< 0.001
**Upper limb pain**						
Number with upper limb pain since before the COVID-19 outbreak	2609	667	510	266	184	
Number with upper limb pain developed during the COVID-19 pandemic	215	66	80	62	40	
**Model 1**		**OR (95% CI)**	**OR (95% CI)**	**OR (95% CI)**	**OR (95% CI)**	
Upper limb pain since before the COVID-19 outbreak vs. without upper limb pain	1	1.64 (1.49–1.80)***	1.82 (1.64–2.03)***	2.69 (2.31–3.13)***	3.18 (2.65–3.81)***	< 0.001
Upper limb pain developed during the COVID-19 pandemic vs. without upper limb pain	1	1.90 (1.44–2.51)***	3.05 (2.34–3.98)***	6.27 (4.65–8.47)***	6.86 (4.79–9.82)***	< 0.001
**Model 2**						
Upper limb pain since before the COVID-19 outbreak vs. without upper limb pain	1	1.51 (1.37–1.67)***	1.68 (1.50–1.88)***	2.20 (1.87–2.57)***	2.44 (2.01–2.96)***	< 0.001
Upper limb pain developed during the COVID-19 pandemic vs. without upper limb pain	1	1.61 (1.21–2.14)**	2.51 (1.91–3.31)***	4.31 (3.13–5.93)***	4.70 (3.22–6.86)***	< 0.001
**Low back pain**						
Number with low back pain since before the COVID-19 outbreak	5673	1252	869	426	267	
Number with low back pain developed during the COVID-19 pandemic	257	66	88	69	54	
**Model 1**		**OR (95% CI)**	**OR (95% CI)**	**OR (95% CI)**	**OR (95% CI)**	
Low back pain since before the COVID-19 outbreak vs. without low back pain	1	1.48 (1.37–1.60)***	1.38 (1.26–1.51)***	1.97 (1.72–2.25)***	2.09 (1.77–2.48)***	< 0.001
Low back pain developed during the COVID-19 pandemic vs. without low back pain	1	1.69 (1.28–2.22)***	2.78 (2.16–3.57)***	6.02 (4.52–8.02)***	7.98 (5.79–11.00)***	< 0.001
**Model 2**						
Low back pain since before the COVID-19 outbreak vs. without low back pain	1	1.41 (1.30–1.53)***	1.39 (1.27–1.53)***	1.85 (1.61–2.13)***	1.85 (1.55–2.21)***	< 0.001
Low back pain developed during the COVID-19 pandemic vs. without low back pain	1	1.42 (1.08–1.88)*	2.30 (1.77–2.99)***	4.11 (3.03–5.59)***	5.48 (3.90–7.70)***	< 0.001
**Leg pain**						
Number with leg pain since before the COVID-19 outbreak	2943	705	460	237	179	
Number with leg pain developed during the COVID-19 pandemic	178	49	57	52	41	
**Model 1**		**OR (95% CI)**	**OR (95% CI)**	**OR (95% CI)**	**OR (95% CI)**	
Leg pain since before the COVID-19 outbreak vs. without leg pain	1	1.57 (1.43–1.73)***	1.52 (1.36–1.70)***	2.29 (1.96–2.68)***	3.14 (2.60–3.77)***	< 0.001
Leg pain developed during the COVID-19 pandemic vs. without leg pain	1	1.75 (1.27–2.40)***	2.83 (2.08–3.85)***	7.19 (5.17–9.99)***	10.14 (7.04–14.61)***	< 0.001
**Model 2**						
Leg pain since before the COVID-19 outbreak vs. without leg pain	1	1.45 (1.32–1.60)***	1.39 (1.24–1.57)***	1.86 (1.58–2.20)***	2.46 (2.102–2.99)***	< 0.001
Leg pain developed during the COVID-19 pandemic vs. without leg pain	1	1.51 (1.09–2.10)***	2.20 (1.60–3.03)***	4.91 (3.45–6.98)***	6.83 (4.63–10.09)***	< 0.001

Model 1: Adjusted for age and sex.

Model 2: Adjusted for age, sex, educational level, marital status, living alone, employment status, equivalized income, smoking status, alcohol consumption, physical activity, sleep duration, history of depression, and history of mental illnesses other than depression.

*COVID-19* coronavirus disease 2019, *OR* odds ratio, *CI* confidence interval.

Loneliness was measured using the Japanese version of the University of California, Los Angeles Loneliness Scale (Version 3), Short Form 3-item assessment tool.

For loneliness, *P* values were calculated to compare participants without loneliness to those in the other categories by multinomial logistic regression.

For social isolation, *P* values were calculated to compare participants who did not feel increased social isolation during the COVID-19 pandemic at all to those in the other categories by multinomial logistic regression.

*p < 0.05, **p < 0.01, ***p < 0.001. n = 25,482.

### Pain intensity

**Figure 5:**
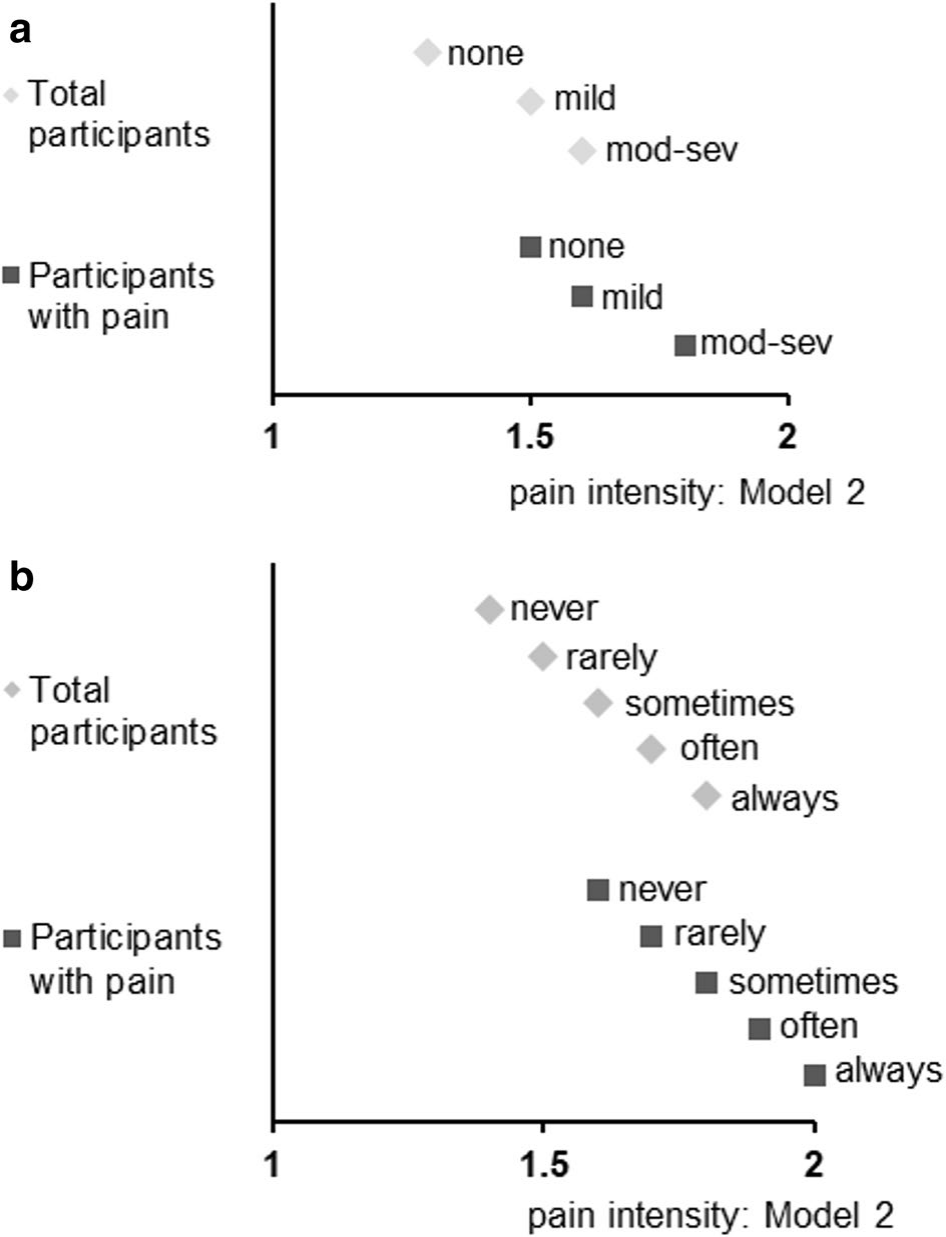
Degree of loneliness/frequency of feelings of social isolation and pain intensity. (**a**) Degree of loneliness in total participants and participants with pain; (**b**) Frequency of feelings of social isolation in total participants and participants with pain. X-axis indicated adjusted mean value of pain intensity ranged 1 to 5 (in model 2). Abbreviation: *mod-sev* moderate-to-severe.

Table 3 and Fig. 5 indicate the differences in reported pain intensity according to the UCLA-LS3-SF3 scoring groups and the frequency with which participants reported an increase in feelings of social isolation. Compared to participants who did not experience loneliness or increased social isolation, those who did reported more severe pain intensity. In Model 2, for individuals who reported any pain symptoms, the adjusted mean pain intensity values among those reporting a lack of loneliness, mild loneliness, and moderate-to-severe loneliness were 1.5, 1.6, and 1.8, respectively; among those reporting increased feelings of social isolation during the COVID-19 pandemic, the adjusted mean pain intensity values for the five frequencies were 1.6, 1.7, 1.8, 1.9, and 2.0.

**Table 3:** The association of loneliness and a feeling of increased social isolation with pain intensity.

	Degree of loneliness										
	None (3 points) n = 14,277		Mild (4–5 points) (n = 3250)		Moderate-to-severe (6–15 points) n = 7955		*P* for trend				
	Adjusted mean	SE	Adjusted mean	SE	Adjusted mean	SE					
**Pain intensity for total participants, n = 25,482**											
Model 1	1.3	0.01	1.5***	0.01	1.7***	0.01	< 0.001				
Model 2	1.3	0.01	1.5***	0.01	1.6***	0.01	< 0.001				
**Pain intensity for participants with any pain symptoms, n = 15,541**											
Model 1	1.5	0.01	1.6***	0.02	1.8***	0.01	< 0.001				
Model 2	1.5	0.01	1.6***	0.01	1.8***	0.01	< 0.001				

	Frequency of feelings of increased social isolation during the COVID-19 pandemic										
	Never n = 18,168		Rarely n = 3188		Sometimes n = 2454		Often n = 1033		Always n = 639		*P* for trend
	Adjusted mean	SE	Adjusted mean	SE	Adjusted mean	SE	Adjusted mean	SE	Adjusted mean	SE	
**Pain intensity total participants, n = 25,482**											
Model 1	1.4	0.01	1.5***	0.01	1.6***	0.01	1.8***	0.02	2.0***	0.03	< 0.001
Model 2	1.4	0.01	1.5***	0.01	1.6***	0.01	1.7***	0.02	1.8***	0.03	< 0.001
**Pain intensity for participants with any pain symptoms, n = 15,541**											
Model 1	1.5	0.01	1.7***	0.02	1.8***	0.02	2.0***	0.03	2.1***	0.03	< 0.001
Model 2	1.6	0.01	1.7***	0.01	1.8***	0.02	1.9***	0.03	2.0***	0.03	< 0.001

Model 1: Adjusted for age and sex.

Model 2: Adjusted for age, sex, educational level, marital status, living alone, employment status, equivalized income, smoking status, alcohol consumption, physical activity, sleep duration, history of depression, and history of mental illnesses other than depression.

*COVID-19* coronavirus disease 2019, *OR* odds ratio, *CI* confidence interval.

Loneliness was measured using the Japanese version of the University of California, Los Angeles Loneliness Scale (Version 3), Short Form 3-item assessment tool.

Pain symptoms were headache, neck/shoulder pain, upper limb pain, low back pain, leg pain, or the presence of chronic pain.

*P* values were calculated to compare participants who without loneliness / did not feel increased social isolation during the COVID-19 pandemic at all to those in the other categories using the analysis of covariance with Dunnett's test.

***p < 0.001. n = 25,482.

### History of past and present chronic pain

**Figure 6:**
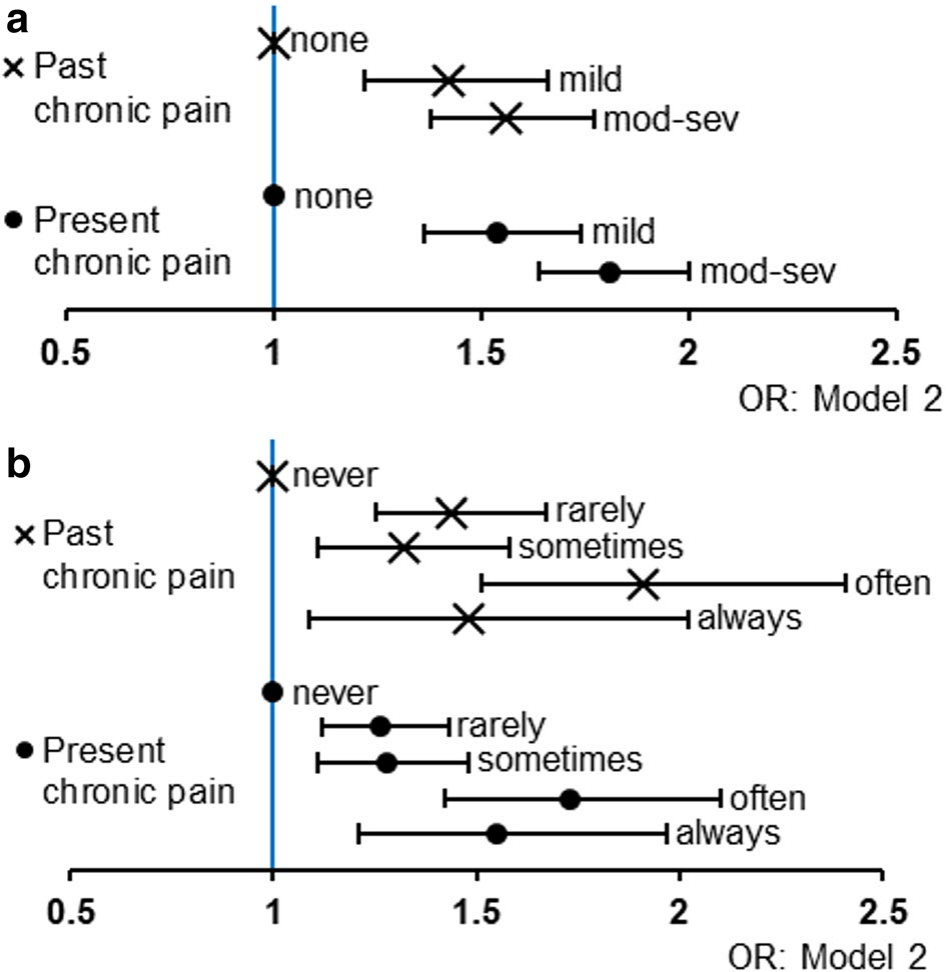
Loneliness/social isolation and history of past/present chronic pain. (**a**) Odds ratios of the prevalence of past and present chronic pain in model 2 by the degree of loneliness; (**b**) Odds ratios of the prevalence of past and present chronic pain in model 2 according to the frequency of feelings of social isolation. X-axis indicated odds ratio. Bars indicated 95% confidence intervals. Abbreviations: *mod-sev* moderate-to-severe; *OR* odds ratio.

Table 4 and Fig. 6 indicate that loneliness and a feeling of increased social isolation were both positively associated with the history of past and present chronic pain.

**Table 4:** The association of loneliness and a feeling of increased social isolation with a history of past and present chronic pain during the COVID-19 pandemic.

	Degree of loneliness					
	None 3 points n = 14,277	Mild 4–5 points n = 3250	Moderate-to-Severe 6–15 points n = 7955	*P* for trend		
Number with history of past chronic pain	734	241	622			
Number with history of present chronic pain	1134	396	1118			
**Model 1**		**OR (95% CI)**	**OR (95% CI)**			
History of past chronic pain vs. without history of chronic pain	1	1.60 (1.37–1.86)***	2.02 (1.80–2.27)***	< 0.001		
History of present chronic pain vs. without history of chronic pain	1	1.71 (1.51–1.93)***	2.44 (2.23–2.68)***	< 0.001		
**Model 2**						
History of past chronic pain vs. without history of chronic pain	1	1.42 (1.22–1.66)***	1.56 (1.38–1.77)***	< 0.001		
History of present chronic pain vs. without history of chronic pain	1	1.54 (1.36–1.74)***	1.81 (1.64–2.00)***	< 0.001		

	Frequency of feeling of increased social isolation during the COVID-19 pandemic					
	Never n = 18,168	Rarely n = 3188	Sometimes n = 2454	Often n = 1033	Always n = 639	*P* for trend
Number with history of past chronic pain	1007	265	170	103	52	
Number with history of present chronic pain	1707	392	282	163	104	
**Model 1**		**OR (95% CI)**	**OR (95% CI)**	**OR (95% CI)**	**OR (95% CI)**	
History of past chronic pain vs. without history of chronic pain	1	1.69 (1.47–1.95)***	1.54 (1.30–1.83)***	2.65 (2.12–3.30)***	2.14 (1.59–2.88)***	< 0.001
History of present chronic pain vs. without history of chronic pain	1	1.48 (1.32–1.67)***	1.55 (1.35–1.78)***	2.47 (2.07–2.94)***	2.63 (2.11–3.29)***	< 0.001
**Model 2**						
History of past chronic pain vs. without history of chronic pain	1	1.44 (1.25–1.67)***	1.32 (1.11–1.58)**	1.91 (1.51–2.41)***	1.48 (1.09–2.02)**	< 0.001
History of present chronic pain vs. without history of chronic pain	1	1.26 (1.12–1.43)**	1.28 (1.11–1.48)*	1.73 (1.42–2.10)***	1.55 (1.21–1.97)*	< 0.001

Model 1: Adjusted for age and sex.

Model 2: Adjusted for age, sex, educational level, marital status, living alone, employment status, equivalized income, smoking status, alcohol consumption, physical activity, sleep duration, history of depression, and history of mental illnesses other than depression.

*COVID-19* coronavirus disease 2019, *OR* odds ratio, *CI* confidence interval.

Loneliness was measured using the Japanese version of the University of California, Los Angeles Loneliness Scale (Version 3), Short Form 3-item assessment tool.

For loneliness, *P* values were calculated to compare participants without loneliness to those in the other categories by multinomial logistic regression.

For social isolation, *P* values were calculated to compare participants who did not feel increased social isolation during the COVID-19 pandemic at all to those in the other categories by multinomial logistic regression.

*p < 0.05, **p < 0.01, ***p < 0.001. n = 25,482.

## Discussion

The predominant location where participants reported experiencing pain during the COVID-19 pandemic was the neck or shoulders, followed by low back pain. Of the total number of participants, 6.3% reported a history of past chronic pain, and 10.4% were presently experiencing chronic pain. Both loneliness and a perception of increased social isolation during the COVID-19 pandemic were positively associated with the prevalence/incidence of all types of pain, pain intensity, and the prevalence of past/present chronic pain in a dose–response manner.

These results were consistent with previous studies which investigated the negative effects of loneliness and social isolation with pain^10,11,13,14^. However, the association between social isolation and a higher risk of pain was partly contrary to a previous study that reported that people who experienced chronic musculoskeletal pain had a higher risk of loneliness but had a lower risk of being socially isolated^12^. They measured structural social isolation (e.g., marital/cohabiting status, social contact, and social participation) using a questionnaire^12^, which was different from the social isolation we used, which was similar to the awareness of social loneliness. This difference in the assessment of social isolation may induce different results. While they assumed that people with musculoskeletal pain might increase in contact with family and friends or social networks in support of taking them to healthcare appointments, people with pain symptoms in the present study may find it difficult to contact or make social network-related healthcare appointments during the COVID-19 pandemic.

To the best of our knowledge, the present study is the first to provide direct evidence of the impact that loneliness and the perception of increased social isolation during the COVID-19 pandemic has had on individuals with pain and a prior history of chronic pain. We assumed several underlying mechanisms that could explain these findings.

In general, loneliness and social isolation are factors associated with psychological stress and distress that can induce depressive symptoms^30,31^, and a previous meta-analysis revealed that loneliness had a moderate impact on depression^6^. Depressive symptoms have been shown to be comorbid with pain and increased pain intensity^32,33^; in turn, loneliness and social isolation would be associated with increased pain responses or perceptions mediated by depressive symptoms, even in the absence of an actual COVID-19 infection.

As a biophysiological mechanism, social isolation may change the threshold of pain sensitivity^34^. In animal models, social isolation in rodents has been shown to result in changed pain thresholds^34,35^; for example, in group-housed mice with immobilization stress, increased pain thresholds have been reported following morphine administration, although this effect was not observed in socially isolated mice^34^. Thus, social isolation due to the COVID-19 pandemic may change the perception of pain in humans, as increased pain intensity could be mediated by a change in the perception of pain.

A previous cohort study investigated the impact of a catastrophic event on pain responses in humans; the study examined those with pain before and approximately six months after the September 11th terrorist attacks in New York and New Jersey, showing that residents reported an increased prevalence of musculoskeletal pain, but not pain from fibromyalgia, after the terrorist attacks^36^. Although the impact of a terrorism event on health may differ from that of a pandemic, and although we did not investigate fibromyalgia, the results of the present study are consistent with the reported changes in musculoskeletal pain.

The findings of the present study indicate that it is important for healthcare providers and policymakers to pay attention to the increased loneliness and social isolation experienced during the COVID-19 pandemic because they may be associated with an increased incidence and worsening of pain symptoms, and the changes may be exacerbated in individuals who have a history of present chronic pain.

A strength of the present study was that it included a large, population-based sample of approximately 25,000 individuals, providing sufficient statistical power for analysis. The present study had several limitations, however. First, we could not determine whether individual participants had been diagnosed with COVID-19 or not. An actual COVID-19 infection could be a potential cofounder in the present study, as individuals who were COVID-19-positive would have been required to adhere to strict social isolation procedures over a couple of weeks. Second, we examined participants’ perceptions of increased social isolation and did not objectively examine the impact of this social isolation on pain (e.g., based on geographical conditions and events). Objective details about social isolation could provide additional useful information. Third, the web-based survey may have biased the distribution and the present study may not be truly representative of the general population, although the demographic profile of the participants was consistent with the official Japanese demographic composition based on the Japanese Vital Statistics for age, sex, and living area as of October 1, 2019^37^. The current prevalence of chronic pain (pain duration ≥ 3 months; 10.4%) was one-quarter of that described in a previous report (39.3%) that was based on a postal survey of Japanese adults, aged 20 years or older, living in a single city^26^. The prevalence of present chronic pain reported in previous web-based surveys was approximately 20%, although these studies were limited by the fact that they only assessed those with moderate-to-severe pain intensity (numerical rating scale score of ≥ 5 out of 10)^27,28^. This discrepancy may be a result of the fact that the present study included more healthy individuals compared with previous surveys. In addition, the participants who were recruited in previous surveys may have had a more particular interest in pain compared with the general population; therefore, the current study population may have been more representative than those of previous reports. Finally, there may have been some recall bias in this study, as it used a cross-sectional design; thus, temporal aspects cannot be discussed. For example, individuals experiencing pain who felt increased social isolation following the COVID-19 outbreak may be more likely to recall the onset of their pain as being related to the pandemic. Future studies will need to clarify this using a prospective design.

In conclusion, in accordance with their severity, loneliness and the perception of increased social isolation during the COVID-19 pandemic were positively associated with the prevalence/incidence of pain, pain intensity, and the prevalence of past and present chronic pain. These findings suggest that loneliness and social isolation should be considered, especially as they relate to pain experienced during the COVID-19 pandemic period.

## Supplementary Information

Supplementary Information.

Supplementary Table 1.

## Data Availability

The data used in this study are not available in a public repository because they contain personally identifiable or potentially sensitive patient information. Based on the regulations for ethical guidelines in Japan, the Research Ethics Committee of the Osaka International Cancer Institute has imposed restrictions on the dissemination of the data collected in this study. All data enquiries should be addressed to the person responsible for data management, Dr. Takahiro Tabuchi at the following e-mail address: tabuchitak@gmail.com.
