# Supplementary material for: Loneliness, social isolation, and pain following the COVID-19 outbreak: data from a nationwide internet survey in Japan
Source: Sci Rep. 2021 Sep 20;11:18643. doi: 10.1038/s41598-021-97136-3 (PMC8452720; doi:10.1038/s41598-021-97136-3)
Supplement: Supplementary file 1 — Supplementary Information. [file 41598_2021_97136_MOESM1_ESM.pdf]

Supplementary methods: the internet survey

Participants in the Japan COVID-19 and Society Internet Survey (JACSIS) were recruited by e-mail invitation from panelists who had been registered with a Japanese internet survey agency (Rakuten Insight, Inc., Tokyo, Japan https://in.m.aipsurveys.com). The survey agency has previously described their methods of quality control for the sampling of panelists [1]. The invitation was sent to candidates who were randomly selected from approximately 2.2 million panelists using a computer algorithm from August 25, 2020 to September 30, 2020 (37 days); this selection was consistent with the official Japanese demographic composition as of October 1, 2019 described by the Japanese Vital Statistics for each category of age, sex, and living area (i.e., prefecture) [2]. Candidates responded to the web-based questionnaire if they agreed to provide web-based informed consent and intended to participate in the JACSIS.

A detailed sampling method and participation rate

The targeted final sample size (n=28,000) in the present internet survey was determined based on a statistical presumption (we needed sufficient numbers in each age and sex stratum to estimate the proportion of events) and the available budget for the survey.

The participation rate is defined as the number of respondents who have provided an eligible response, divided by the total number of initial personal invitations requesting participation [3]. In the JACSIS, the participation rate was defined as the proportion of the number of participants relative to the final number of invitations. A total of 224,389 invitations were delivered; thus, the participation rate in the JACSIS was 28,000/224,389 (12.5%).

The internet survey agency was unable to know whether each candidate received and/or recognized the invitation e-mail or not, and only the final number of participants in the survey was available. Thus, to estimate the collection rate for monitoring purposes, the internet survey agency delivered 28,000 invitations repeatedly until reaching the target of 28,000 participants; this collection rate (the proportion of participants who responded relative to the 28,000 invitations) was identified for each delivery across the first 10 days. At the first invitation (three days after the first delivery), the rate was 12,184/28,000 (43.5%); at the second invitation (five days after the first delivery), the rate was 1,084/28,000 (3.8%); at the third invitation (seven days after the first delivery), the rate was 656/28,000 (2.3%); at the fifth invitation (10 days after the first delivery, the rate was 606/28,000 (2.2%); the cumulative collection rate was 14,530/28,000 (51.9%) over the first 10 days. Technically, the method was skewed toward those who had participated in the survey earlier, and the apparent participation rate of internet surveys is likely to be lower than paper-based surveys administered on-site.

1. Rakuten Insight, Inc., Tokyo, Japan. Policies. 2019 (Last updated: Feb 28, 2019). Available:

https://in.m.aipsurveys.com/policies. Accessed 11 Nov 2020.

2. The Ministry of Health Labour and Welfare. Vital Statistics. 2019. Available: https://www.e-stat.go.jp/en/stat-search/files?page=1&toukei=00450011&tstat=000001028897. Accessed 12 Nov 2020.

3. American Association for Public Opinion Research. Standard Definitions: Final Dispositions of Case Codes and Outcome Rates for Surveys 8th edition. In. 2015.
