# Supplementary material for: Loneliness, social isolation, and pain following the COVID-19 outbreak: data from a nationwide internet survey in Japan
Source: Sci Rep. 2021 Sep 20;11:18643. doi: 10.1038/s41598-021-97136-3 (PMC8452720; doi:10.1038/s41598-021-97136-3)
Supplement: Supplementary file 2 — Supplementary Table 1. [file 41598_2021_97136_MOESM2_ESM.pdf]

Supplementary Table 1. Participant characteristics (n=25,482)

	n	(%)
**Age, years**		
15–19	1,214	(4.8)
20–29	3,211	(12.6)
30–39	3,767	(14.8)
40–49	4,894	(19.2)
50–59	4,256	(16.7)
60–69	4,243	(16.7)
70–79	3,897	(15.3)
**Sex**		
Women	12,809	(50.3)
Men	12,673	(49.7)
**Body mass index, kg/m2**		
< 18.5	2,993	(11.7)
18.5 –24.9	17,579	(69.0)
25.0–29.9	4,036	(15.8)
≥ 30.0	874	(3.4)
**Educational level**		
Less than high school	1,014	(4.0)
High school	8,375	(32.9)
Vocational school	2,677	(10.5)
Junior or technical college	2,582	(10.1)
University	9,705	(38.1)
Graduate school	1,067	(4.2)
Others	62	(0.2)
**Marital status**		
Married or common law	15,230	(59.8)
Single	7,806	(30.6)
Divorced	1,602	(6.3)
Widowed	844	(3.3)
**Living alone**		
Yes	4,997	(19.6)
No	20,485	(80.4)
**Employment status**		
Company executive	847	(3.3)
Owner of family operated business	1,449	(5.7)
Employee of family operated business	196	(0.8)

Management level employee	1,887	(7.4)
Full-time employee	6,779	(26.6)
Contract employee	1,338	(5.3)
Part-time employee/on-the-side worker	2,958	(11.6)
Student	1,751	(6.9)
Retired	1,065	(4.2)
Full-time homemaker	4,197	(16.5)
Unemployed	3,015	(11.8)
**Equivalized household income, million Japanese yen**		
Q1: –1.82	4,303	(16.9)
Q2: 1.83–2.64	3,884	(15.2)
Q3: 2.65–3.75	3,862	(15.2)
Q4: 3.76–5.306	4,197	(16.5)
Q5: 5.307–20.00	3,962	(15.5)
Missing	5,274	(20.7)
**Smoking status**		
Never-smoker	13,917	(54.6)
Ex-smoker	6,984	(27.4)
Smoker	4,581	(18.0)
**Alcohol consumption**		
Never	5,537	(21.7)
Ex-drinker	7,788	(30.6)
Social drinker	6,860	(26.9)
Drinker: < 23 g	1,197	(4.7)
Drinker: 23–45 g	1,956	(7.7)
Drinker: ≥ 46 g	2,144	(8.4)
**Amount of physical activity after COVID-19 outbreak**		
Decreased	7,358	(28.9)
No changed	15,677	(61.5)
Increased	2,447	(9.6)
**Sleep duration**		
< 4 hours	2,297	(9.0)
≥ 4 hours, < 6 hours	4,342	(17.0)
≥ 6 hours, < 8 hours	13,993	(54.9)
≥ 8 hours, < 10 hours	3,575	(14.0)
≥ 10 hours	301	(1.2)
Hard to respond/Unsure	974	(3.8)
**History of depression**		

None	23,128	(90.8)
Have a history of depression but already recovered	1,385	(5.4)
Yes, receiving treatment	694	(2.7)
Yes, without treatment	275	(1.1)
**History of mental illnesses other than depression**		
None	23704	(93.0)
Have a history of mental illnesses but already recovered	828	(3.2)
Yes, receiving treatment	673	(2.6)
Yes, without treatment	277	(1.1)
	**Means**	**(SD)**
**UCLA-LS3-SF3 score (3-15 points)**	5.1	(3.2)
	**n**	**(%)**
**Frequency of feeling of increased social isolation during the COVID-19 pandemic**		
Never	18168	(71.3)
Rarely	3188	(12.5)
Sometimes	2454	(9.6)
Often	1033	(4.1)
Always	639	(2.5)
**Headache**		
None	18,985	(74.5)
Yes, since before COVID-19 outbreak	5,987	(23.5)
Yes, developed during COVID-19 pandemic	510	(2.0)
**Neck or shoulder pain**		
None	14,077	(55.2)
Yes, since before the COVID-19 outbreak	10,803	(42.4)
Yes, developed during the COVID-19 pandemic	602	(2.4)
**Upper limb pain**		
None	20,783	(81.6)
Yes, since before the COVID-19 outbreak	4,236	(16.6)
Yes, developed during the COVID-19 pandemic	463	(1.8)
**Low back pain**		
None	16,461	(64.6)
Yes, since before the COVID-19 outbreak	8,487	(33.3)
Yes, developed during the COVID-19 pandemic	534	(2.1)
**Leg pain**		
None	20,581	(80.8)
Yes, since before COVID-19 the outbreak	4,524	(17.8)

Yes, developed during COVID-19 the pandemic	377	(1.5)
**Pain intensity**		
(1) No pain or discomfort	16,556	(65.0)
(2) Slight pain or discomfort	7,144	(28.0)
(3) Moderate pain or discomfort	1,263	(5.0)
(4) Severe pain or discomfort	391	(1.5)
(5) Extreme pain or discomfort	128	(0.5)
**Chronic pain**		
None	21,237	(83.3)
Have a history of chronic pain but have already recovered	1,597	(6.3)
Yes, receiving treatment	1,000	(3.9)
Yes, without treatment	1,648	(6.5)

Abbreviations: SD, standard deviation; Q, Quintile; COVID-19, coronavirus disease 2019; UCLA-LS3-SF3, the University of California, Los Angeles Loneliness Scale (Version 3), Short Form 3-item.
